# Scalable evaluation framework for retrieval augmented generation in tobacco research using large Language models

**DOI:** 10.1038/s41598-025-05726-2

**Published:** 2025-07-02

**Authors:** Sherif Elmitwalli, John Mehegan, Sophie Braznell, Allen Gallagher

**Affiliations:** Tobacco Control Research Group, Department for Health, University of Bath, Bath, UK

**Keywords:** Retrieval-Augmented generation, Domain-Specific information retrieval, AI evaluation, Goal-Question-Metric framework, Large Language models, Expert validation, Computer science, Software, Public health

## Abstract

Retrieval-augmented generation (RAG) systems show promise in specialized knowledge domains, but the tobacco research field lacks standardized assessment frameworks for comparing different large language models (LLMs). This gap impacts public health decisions that require accurate, domain-specific information retrieval from complex tobacco industry documentation. To develop and validate a tobacco domain-specific evaluation framework for assessing various LLMs in RAG systems that combines automated metrics with expert validation. Using a Goal-Question-Metric paradigm, we evaluated two distinct LLM architectures in RAG configurations: Mixtral 8 × 7B and Llama 3.1 70B. The framework incorporated automated assessments via GPT-4o alongside validation by three tobacco research specialists. A domain-specific dataset of 20 curated queries assessed model performance across nine metrics including accuracy, domain specificity, completeness, and clarity. Our framework successfully differentiated performance between models, with Mixtral 8 × 7B significantly outperformed Llama 3.1 70B in accuracy (8.8/10 vs. 7.55/10, p < 0.05) and domain specificity (8.65/10 vs. 7.6/10, p < 0.05). Case analysis revealed Mixtral’s superior handling of industry-specific terminology and contextual relationships. Hyperparameter optimization further improved Mixtral’s completeness from 7.1/10 to 7.9/10, demonstrating the framework’s utility for model refinement. This study establishes a robust framework specifically for evaluating LLMs in tobacco research RAG systems, with demonstrated potential for extension to other specialized domains. The significant performance differences between models highlight the importance of domain-specific evaluation for public health applications. Future research should extend this framework to broader document corpora and additional LLMs, including commercial models.

## Introduction

The rapid advancement of artificial intelligence (AI) and natural language processing (NLP) technologies has profoundly transformed the landscape of information retrieval and knowledge management^1^. These innovations are particularly impactful in fields that require the efficient processing and utilization of vast, domain-specific knowledge. One such critical domain is tobacco-related research, which encompasses a wide array of topics, including public health, regulatory policies, industry practices, and scientific studies on the health impacts of tobacco use^2^. The effective extraction of relevant information from the extensive and complex corpus of tobacco-related documents—ranging from peer-reviewed publications and industry reports to legal frameworks and public health guidelines—is essential for researchers, policymakers, and public health professionals. These challenges are compounded by the need to synthesize diverse data formats, understand the nuanced language used across public health and industry contexts, and ensure accuracy in summarizing both quantitative and qualitative information^3^. Addressing these issues requires the development of more sophisticated AI-driven approaches tailored to the evolving demands of tobacco research^4,5^.

In recent years, machine learning (ML) has shown considerable promise in addressing some of these challenges within tobacco research. The applications of ML in this domain include smoking cessation support, social media content analysis, and predictive modelling of tobacco-related health outcomes^6,7^. Despite these advances, traditional information retrieval methods often fall short in handling the nuanced and context-dependent nature of queries within this highly specialized field^8^. These limitations can lead to incomplete or irrelevant results, which in turn can hinder effective decision-making and policy development, as shown^10^. As the field of tobacco research continues to evolve rapidly, there is a pressing need for more advanced tools that can keep pace with the growing body of knowledge and provide accurate, timely insights^9^.

Neural models, particularly those powered by deep learning, have begun to address some of these limitations. The availability of large datasets and advancements in computational power have enabled the development of sophisticated models capable of processing and interpreting complex information^10^. However, these models often require substantial data and computational resources and may still struggle to fully capture the semantic depth of queries in domains where the knowledge base is both vast, constantly evolving and nuanced. In such fields, the ability to integrate and update information in real-time is critical, making traditional static models increasingly inadequate for the task^11^.

To overcome these challenges, RAG systems have emerged as promising solutions. RAG systems combine the generative capabilities of large language models (LLMs) with the precision of domain-specific knowledge bases, enabling them to retrieve and synthesize information more effectively than traditional retrieval methods do. By leveraging the strengths of both retrieval and generation, RAG systems can provide more accurate and contextually relevant responses to complex queries, making them particularly useful in specialized domains such as tobacco research, where the need for accurate information is high^12,13^.

Despite the potential of RAG systems, there is a significant gap in the literature concerning their evaluation and optimization in domain-specific contexts. Most existing studies on RAG systems have focused on open-domain question-answering tasks, where the challenges of domain specificity and contextual relevance are less pronounced^14^. The application of RAG systems to specialized domains such as tobacco research remains underexplored, and there is a need for more systematic approaches to evaluate their performance in these contexts. Traditional evaluation metrics, which are often designed for general-purpose models, may not fully capture the nuanced requirements of specialized domains. Moreover, manual evaluation processes are not only time-consuming and labour-intensive but also subject to inconsistencies, making them less reliable for ongoing optimization efforts^15,16^.

To address these challenges, our research introduces a novel framework for evaluating and optimizing RAG systems tailored specifically to the domain of tobacco research. This framework leverages advanced language models not only for information retrieval and generation but also as tools for systematically evaluating the RAG system itself. A key component of this approach is the development of human-verified reference responses, which serve as the gold reference for consistently evaluating the performance of the core LLMs within the RAG system. The evaluation framework is further enhanced by the integration of a Goal-Question-Metric (GQM) approach, which systematically assesses the RAG system across various dimensions, including relevance, accuracy, completeness, and scalability. By establishing these reference responses and applying the GQM methodology, we aim to create a reliable benchmark that allows for the rigorous comparison and optimization of different RAG configurations, ensuring that the system meets the specific needs of tobacco research.

GQM provides a structured framework for defining and analysing measurement goals. It operates on three hierarchical levels: at the conceptual level, the goal defines the purpose of measurement from specific perspectives; at the operational level, questions are formulated to characterize the assessment process; and at the quantitative level, metrics are identified to provide measurable answers to these questions. The evaluation framework employs nine carefully selected metrics that address critical aspects of information retrieval and generation in tobacco research. These metrics are essential because tobacco research demands high standards of accuracy when dealing with regulatory requirements and industry practices, completeness in covering complex relationships between scientific evidence and policy implications, and precision in using domain-specific terminology. Clear and coherent presentation of information is vital for policy decisions, while context awareness ensures appropriate interpretation of industry documentation. Adaptability and domain specificity are particularly important given the diverse nature of tobacco research queries, from scientific assessments to market analyses. Together, these metrics provide a comprehensive evaluation framework that aligns with the specific needs of tobacco research applications.

In fields such as software development, GQM has been crucial for understanding system performance, identifying improvement strategies, and creating objective evaluation criteria^17^. For example, in software quality assessment, researchers use GQM to define goals such as improving software reliability, formulating questions about code complexity and error rates, and developing metrics to quantitatively measure these aspects. Its adaptability makes GQM particularly powerful in complex domains where traditional evaluation methods may fall short, offering a flexible yet rigorous approach to assessment that can be tailored to the specific nuances of different research contexts^18^.

To address the gaps identified in domainspecific RAG evaluation, the work is structured around three research questions:

RQ1: How do Mixtral 8 × 7B and Llama 3.1 70B differ in their ability to retrieve and generate accurate, contextaware responses to tobacco research queries that involve industry-specific terminology, regulatory concepts, and scientific information?

RQ2: Which of the nine-evaluation metrics (relevance, accuracy, completeness, clarity, conciseness, coherence, context awareness, adaptability, domain specificity) show the largest performance gaps between these two model architectures?

RQ3: To what extent can hyperparameter optimization enhance the performance of a domainspecific RAG configuration in tobacco research?

Answering these questions, this paper makes four key contributions:

1. The extension of the GoalQuestionMetric paradigm to RAG systems by defining a structured, tobaccotailored mapping of goals, questions, and quantitative metrics, thereby advancing theory in AI evaluation.
2. The development and public release of a goldstandard dataset of 20 specialistvalidated tobacco query–response pairs, enabling reproducible, expertgrounded benchmarking of RAG configurations and providing a valuable resource for tobacco researchers to understand how language models interpret industry documentation.
3. We provide the first headtohead comparison of Mixtral 8 × 7B versus Llama 3.1 70B in a tobacco domain RAG setting, demonstrating statistically significant performance differences across all nine metrics.
4. We demonstrate how hyperparameter tuning (embedding model, chunk size/overlap, retrieval count) can meaningfully boost RAG performance offering practical guidance for system refinement.

Finally, we show that our evaluation framework is modelagnostic and readily adaptable to other specialized fields (e.g. healthcare, legal research, environmental policy), supporting scalable, transparent, and rigorous assessment of RAG systems across diverse domains. The framework’s design enables efficient evaluation of any number of LLMs without modification to the core methodology.

## Related work

### Challenges in domainspecific information retrieval

The challenges in domain-specific information retrieval (IR) are multifaceted, including the need for context sensitivity, semantic understanding, and the ability to process vast amounts of specialized information. Rateria and Singh noted that traditional search techniques, which lack these capabilities, often fail to retrieve pertinent data efficiently in large-scale enterprises where extensive textual information is shared across corporate repositories and intranet websites^19^. Wu et al.^20^ showed that domainspecific IR is often undermined by acronyms, abbreviations and typographical errors common in realworld corpora, which traditional stringmatching methods fail to address. They introduced Smash, a dynamic programming–based similarity measure that boosts max and mean Fscores by 23.5% and 110.8%, respectively, without relying on predefined synonym rules. This work underscores the need for robust, domaintailored stringmatching techniques to improve retrieval precision in specialized IR tasks. Tamine and Goeuriot highlighted that despite the explosive growth and accessibility of medical information on the internet leading to increased research activity, current medical search systems exhibit low levels of performance, especially when tackling complex tasks such as searching for diagnoses or treatments^21^. These shortcomings in string-matching and context sensitivity motivate the exploration of neural, retrieval‐augmented approaches, as surveyed in the next subsection.

### Large language models and RAG systems

While domain-specific IR methods struggle with semantic ambiguity and scale, LLMs - especially when combined with retrieval - hold promise. Recent advancements in LLMs have revolutionized the field of artificial intelligence, offering unprecedented performance in various applications. Yang et al. emphasized that LLMs demonstrate remarkable abilities in understanding, language synthesis, and commonsense reasoning, often achieving humanlike performance^22^. In the queryfocused summarization domain, Zhang et al.^23^ propose a novel knowledgeintensive approach that reframes QFS (Query-Focused Summarization) as a twostage task: a retrieval module that identifies potentially relevant documents from a largescale corpus based on the input query, and a summarization controller that leverages an LLM with a tailored prompt to produce comprehensive, queryrelevant summaries. Evaluated on a newly created dataset with humanannotated relevance labels, their method outperforms traditional approaches—especially in scenarios where no preselected document set is available. Building on this foundation, Wiratunga et al. demonstrated the potential of RAG systems in legal questionanswering tasks, highlighting how the integration of casebased reasoning can enrich LLM queries with contextually relevant cases^24^. Despite impressive RAG capabilities, systematic, domain-aware evaluation remains underdeveloped.

### Evaluation frameworks for language models

As LLMs and RAG systems continue to evolve and play vital roles in research and daily use, their evaluation becomes increasingly critical. Chang et al. emphasized the need for comprehensive evaluation methods that assess LLMs not only at the task level but also at the societal level to better understand their potential risks and impacts^25^. Standard IR/NLG (Natural Language Generation) metrics such as ROUGE often fail to reflect true quality in domainspecific summarization tasks, as evidenced by a recent evaluation of five LLMs on clinical dialogues: ROUGE ranked a finetuned transformer highest, yet clinician ratings and the UniEval metric both favored ChatGPT, with UniEval showing strong correlation with expert scores^26^. This underscores the need for domainaware evaluation frameworks that integrate specialized automated measures and humanintheloop assessment^26^. There is a growing recognition of the need for more nuanced and context-aware evaluation methodologies. The authors provided an extensive review of evaluation methods for LLMs, focusing on three key dimensions: what to evaluate, where to evaluate, and how to evaluate. Their work highlighted the importance of considering a wide range of evaluation tasks, from general natural language processing to specialized applications in medicine, ethics, and education. Although these frameworks address general IR/NLG quality, they rarely target the ethical and terminological demands of tobacco research.

### Applications in tobacco research

The optimization of AI systems for domain-specific applications, particularly in fields such as tobacco research, presents unique challenges and opportunities. Fu et al. highlighted that machine learning represents a powerful tool that could advance research and policy decision-making in tobacco control. However, the effective application of these technologies requires careful adaptation to the specific needs and constraints of the domain^27^.

Wilson and Cook discussed domain adaptation techniques, offering approaches for handling situations where a network is trained on labelled data from a source domain and unlabelled data from a related but different target domain. This is particularly relevant in specialized fields such as tobacco research, where labelled data may be scarce, but large amounts of unlabelled domain-specific data are available^28^.

Moreover, the field of tobacco research presents a unique set of challenges for information retrieval and document analysis. For example, Legg et al. highlighted the tobacco industry’s long history of attempting to influence scientific research and public perception, underscoring the need for robust and unbiased information retrieval systems that can effectively navigate the complex landscape of tobacco-related documents^29^.

In addition, the application of AI in healthcare and specialized research domains raises significant ethical concerns. In regulated domains such as tobacco research, system trustworthiness hinges not just on retrieval accuracy but on fairness, accountability, transparency and ethics (FATE). A recent systematic review of FATE in information retrieval develops clear definitions, surveys approaches and evaluation methodologies and proposes taxonomies to quantify each dimension of trustworthiness^30^.

In the context of social media and health information dissemination, Singhal et al. explored the ethical use of AI and machine learning, focusing on the principles of fairness, accountability, transparency, and ethics (FATE). Their work underscored the importance of developing computational methods and approaches that ensure the responsible application of AI in health-related contexts^31^. These studies underscore both the potential and the unresolved need for domain-specific evaluation.

### Research gaps and opportunities

Despite a growing body of literature on AI technologies in specialized domains, several critical gaps remain in the context of tobacco research. First, there is a lack of domainspecific evaluation frameworks for RAG systems: existing metrics and methodologies often fail to capture the nuanced characteristics of highly specialized tobaccorelated corpora. Second, although machine learning methods have demonstrated potential in tobacco control studies, the systematic application and assessment of RAG systems within this field remain underexplored^32^. Third, ethical considerations—particularly fairness, accountability, transparency and bias mitigation—are insufficiently integrated into tobaccofocused information retrieval pipelines, leaving a risk of perpetuating industrydriven misinformation^29,31^. Fourth, there is an absence of costeffective yet robust evaluation methodologies tailored to RAG performance in domainspecific settings, making it difficult for researchers and practitioners to balance resource constraints with rigorous assessment.

Addressing these research gaps is essential for advancing both methodological innovation and the responsible use of AI in tobacco research. In this study, we therefore develop a comprehensive, costefficient evaluation framework designed specifically for RAG systems analysing tobaccorelated documents; conduct a comparative analysis of leading language models within a RAG setup; optimize retrieval and summarization configurations to enhance accuracy and mitigate bias; and propose a generalizable deployment framework for RAG applications in tobacco research.

## Methods

### Overview

**Fig. 1:**
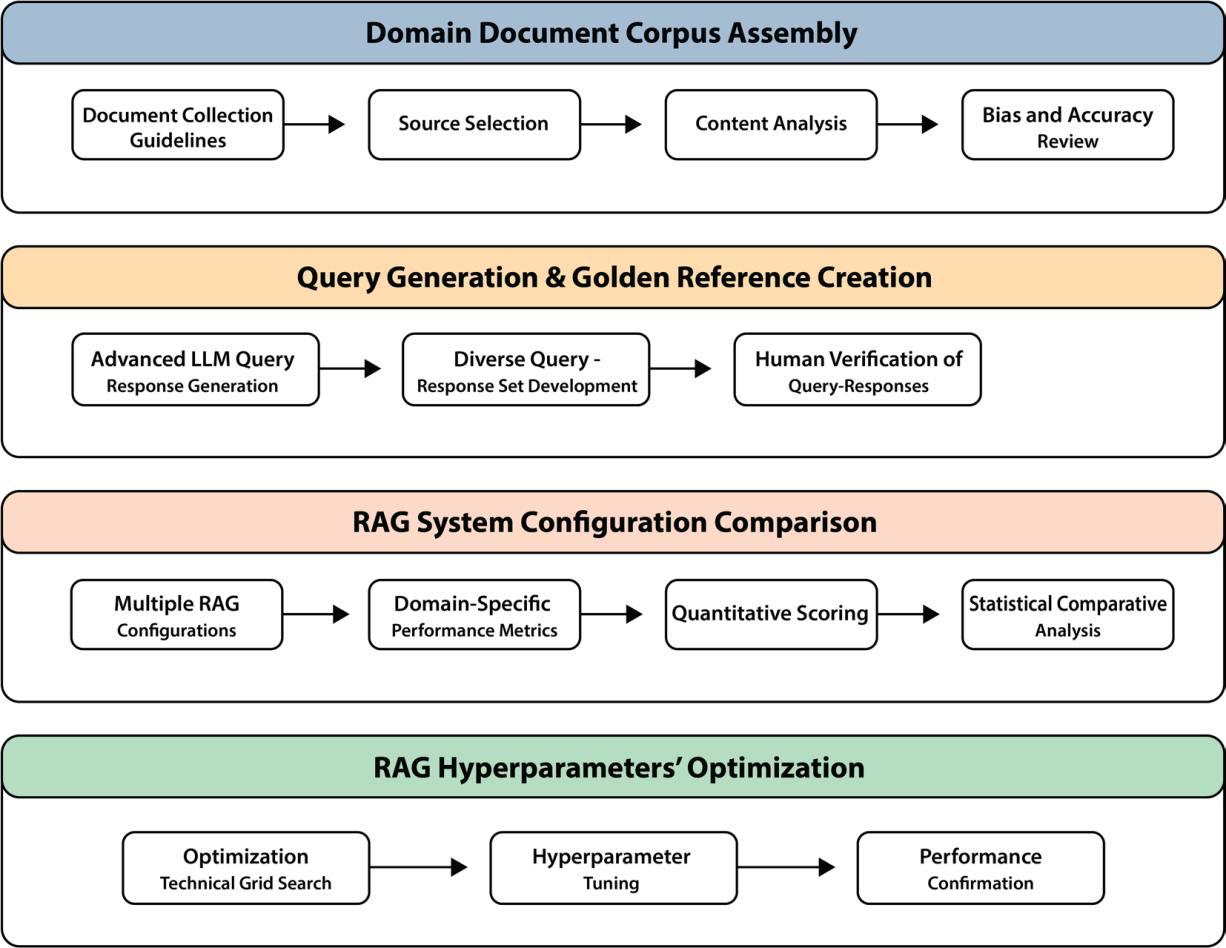
RAG system evaluation framework.

This methodology outlines a detailed approach to evaluating and optimizing RAG systems for tobacco-related document analysis; addresses the complex challenges of domain-specific information retrieval in tobacco research; and emphasizes cost effectiveness, robustness, and adherence to ethical guidelines. The methodology consists of four main phases, each designed to systematically build a robust and reliable evaluation framework, as shown in Fig. 1.

1. The first phase centred on identifying a single, representative document to serve as the foundation for the study. After a thorough evaluation of potential sources, the Philip Morris International (PMI) Integrated Report 2023 was selected for its comprehensive and structured presentation of topics critical to tobacco research from the PMI^33^. This document covers a wide range of essential themes, including business performance, industry strategies, product details, regulatory developments, and health impacts, making it an ideal basis for developing domain-specific queries and responses. The selection process was guided by a rigorous set of criteria to ensure the suitability of the document for the study’s goals. A tobacco domain specialist conducted an in-depth review of potential candidates, assessing each document for content diversity, specificity, and relevance to tobacco research. Particular emphasis was placed on the depth and detail provided by the document, ensuring that it could support the development of complex and nuanced queries. Additionally, the consistency and clarity of the document’s language were evaluated to ensure that it could facilitate precise retrieval and generation tasks within the RAG system.
2. The second phase involves generating and refining queries and reference responses focused on tobacco-related topics, ensuring that they align with the context and content of the chosen dataset, guided by the capabilities of GPT-4o, a language model developed by OpenAI, and domain expertise. We employ a comprehensive query generation strategy that produces a diverse set of questions across various tobacco research topics, guided by prompt engineering and expert input. A taxonomy categorizes these queries by topic, type, and complexity, ensuring a balanced evaluation dataset. This iterative process ensures that the reference responses are accurate, unbiased, and suitable for benchmarking the RAG system.
3. The third phase constitutes the core of our evaluation process. Here, we implement two parallel RAG configurations, one using Mixtral 8 × 7B and another using Llama 3.1 70B as the base language model. These systems process the generated queries, and their outputs are subjected to a multifaceted evaluation. This evaluation employs an innovative approach using the GPT-4o as an automated assessor, guided by a Goal-Question-Metric framework. The automated assessment is calibrated and validated through strategic human expert reviews, ensuring reliability and accuracy in the evaluation process.
4. The fourth phase is based on initial evaluation results; we conduct hyperparameter optimization to maximize the retrieval accuracy. We adjusted parameters such as the embedding model, chunk size, and retrieval count through a grid search, testing each configuration on the gold reference queries. The optimal configuration, identified within the best performing model, was re-evaluated to confirm improvements across metrics such as relevance and domain specificity. This optimized setup provided a reliable and efficient configuration for tobacco-related information retrieval, establishing a benchmark for future model assessments in the RAG framework.

### Preparation and data collection

The foundation of our RAG system evaluation lies in the careful selection and preparation of a relevant and representative tobacco-related document. The PMI 2023 Integrated Report offers a robust foundation for evaluating the RAG system’s ability to handle complex, domain-specific queries. The report not only offers extensive insights into these topics but also provides detailed and specific language that is essential for testing the RAG system’s ability to handle domain-specific queries effectively. Although we did not assemble a broad corpus that includes peer-reviewed scientific literature and public health guidelines, we address potential biases associated with the use of industry-produced materials. Our approach includes an independent review of the content by domain specialists in tobacco research, who critically assess the information to mitigate the risk of biased or misleading representations. This document poses these challenges, which is why it was chosen. Furthermore, we disclose our document choice transparently to ensure that readers are aware of the source of our data and can appropriately interpret our findings within this context.

The pre-processed PMI report was prepared for efficient retrieval via the Chroma vector database. We defined a schema for storing document embeddings and metadata and implemented a scalable indexing strategy tailored to the single-source setup. This design enables rapid and accurate retrieval of information while ensuring that our RAG system can robustly address queries relevant to the document’s content.

Through this focused approach to document selection, including a critical review by domain specialists to assess potential biases and accuracy, we establish a well-defined and transparent foundation for evaluating our RAG system. While narrower in scope than a full corpus, our methodology ensures clarity, precision, and contextual accuracy in the complex domain of tobacco research. The inclusion of a thorough review process ensures that the information drawn from industry-produced materials is critically examined to mitigate risks of bias or misleading representations.

### Query generation and reference response creation

In this step, we developed a precise set of queries and responses tailored to the content of the PMI Report, guided by the expertise of a tobacco domain specialist. The process begins with the primary domain specialist (PS) selecting topics and defining categories of queries on the basis of the document’s key areas. The PS provided guidance on the appropriate number of queries for each topic to ensure balanced representation across essential themes: industry strategies/practices, business/market performance, tobacco/nicotine products, scientific research/health effects, and policy/regulation.

Using these guidelines, an initial set of 20 query‒response pairs was generated via GPT-4o within the RAG system. The primary specialist (PS) carefully reviewed these pairs for accuracy and alignment with the tobacco document, refining them by removing redundant or irrelevant content and adjusting the text to better reflect the document’s details. During this review, it became evident that the “policy/regulation” category was underrepresented due to limited content in the document on this topic. To address this, the PS introduced “language/terminology” as a replacement focus area, ensuring the inclusion of an underexplored dimension of the document. This iterative process enhanced the diversity and relevance of the questions while addressing content gaps. The refined queries underwent further rounds of expert validation to finalize a comprehensive, accurate, and domain-specific reference set. Once this initial review was completed, additional queries were added on certain topics to round out the coverage, ensuring that each topic was represented with multiple well-defined queries.

After the PS finalized a comprehensive set of 20 query‒response pairs, the gold reference set underwent a rigorous secondary review by two additional domain specialists. These specialists used tracked changes to further refine the responses, focusing on factual accuracy, consistency with the PMI Report, and appropriate terminology. This multistage review process allowed for collaborative refinements and ensured that the gold reference set met the highest standards of quality and domain specificity. The iterative validation by multiple specialists not only reinforced the reliability of the reference set but also minimized the risk of bias or inaccuracies, establishing a robust benchmark for evaluating the RAG system’s performance.

Table 1 illustrates the four-stage iterative workflow by which the gold reference of 20 query–response pairs was developed and validated. In the first round, the Primary Tobacco Specialist (PS) drafted an initial set of 14 pairs, removing overly broad queries and aligning responses with the PMI 2023 Integrated Report. In the second round, the PS expanded and refined this set to 17 pairs by introducing underrepresented topic areas—such as industry terminology—and tightening factual accuracy. The third round involved a final PS review to rephrase complex responses for clarity and ensure full alignment with the source document, resulting in a complete set of 20 pairs. Finally, two additional specialists conducted a collaborative review (Round 4), using tracked changes to resolve remaining discrepancies, standardize terminology, and confirm factual precision.

This structured, multireview process guaranteed that each query–response pair met rigorous domainspecific standards. By progressively narrowing focus—from broad topic coverage to precise wording and factual correctness—the table underscores how expert validation and iterative refinement combined to produce a reliable, biasmitigated benchmark for evaluating RAG system outputs in tobacco research.

**Table 1:** Evaluation rounds used to develop the gold standard reference.

Round	Reviewer	Actions taken	Example of changes	Outcome
**1**	Primary Tobacco Specialist	Defined topics, specified query numbers, conducted initial review, removed redundancies, modified responses for specificity.	Removed overly broad queries, ensured responses were tobacco specific.	Produced initial set of 14 pairs.
**2**	Primary Tobacco Specialist	Added queries, refined responses for accuracy and specificity.	Introduced new queries in underrepresented areas, adjusted responses to better align with the PMI document.	Expanded and refined set of 17 pairs.
**3**	Primary Tobacco Specialist	Finalized the initial gold reference by modifying or rejecting responses.	Rephrased complex responses for clarity, ensured responses were factually precise and document aligned.	Prepared set of 20 query-response pairs for subsequent specialist review.
**4**	Second and Third Tobacco Specialist	Assessed the gold reference pairs	Used tracked changes to revise each query-response pairs according to the reference document	Prepared a revised final set of 20 query-response pairs.

### Model selection criteria

Our evaluation focused on comparing two distinct LLM architectures: Mixtral 8 × 7B and Llama 3.1 70B. These models were selected based on several key criteria relevant to real-world deployment in tobacco research contexts:

1. Architectural diversity: Mixtral 8 × 7B employs a mixture-of-experts architecture that theoretically enables more efficient domain specialization, while Llama 3.1 70B represents a traditional dense transformer architecture with larger parameter count. This contrast allows us to examine whether architectural differences impact domain-specific performance.
2. Open accessibility: Both models are openly available, ensuring our framework and findings can be reproduced and extended by other researchers. This accessibility is critical for developing evaluation standards in public health domains where transparency is essential.
3. Resource considerations: Our selection balanced performance potential with computational efficiency. While commercial models like GPT-4o were used as evaluation tools in our framework, they would be prohibitively expensive for deployment as the core RAG model in many research settings. Our framework deliberately focuses on more accessible open-source models that tobacco researchers with varying resource constraints could feasibly implement.
4. Parameter scale comparison: The significant difference in parameter count (Mixtral’s 8 × 7B architecture versus Llama’s 70B parameters) enables investigation of whether larger models necessarily provide better performance in specialized domains.
5. Proof of concept approach: This study demonstrates a proof of concept for our evaluation framework using two representative models with different architectures. The framework itself is designed to be model-agnostic and can be extended to evaluate any number of LLMs, whether open-source or commercial, without modification to the core methodology. It’s important to note that GPT-4o serves a distinct role in our framework as an automated evaluation tool rather than as one of the evaluated models. This separation maintains the integrity of our assessment process while leveraging advanced language understanding capabilities that is associated with human experts validated GPT-4o’s assessments to ensure reliability for consistent evaluation across model outputs.
6. Future extensibility: The framework can incorporate any capable LLM—opensource, commercial (e.g. GPT-4o or its successors), or future releases—can be slotted into either the retrieval/generation or evaluation component without altering the core GQM methodology, ensuring seamless adaptation as new models emerge.

### RAG system evaluation

The RAG system evaluation begins with the setup of two parallel configurations using Mixtral 8 × 7B and Llama 3.1 70B as base language models. Mixtral 8 × 7B, a mixture-of-experts model known for efficiency and task-specific performance, and Llama 3.1 70B, a larger model with broad general language understanding. Moreover, the Mixtral 8 × 7B model is particularly advantageous for scenarios requiring rapid inference and low latency, making it ideal for real-time applications where efficiency is crucial^34^. In contrast, Llama 3.1 70B’s extensive training on diverse datasets equips it with a robust understanding of nuanced language and complex queries, enabling it to generate more contextually relevant and coherent responses across a wide range of topics^35^. This comparison aims to explore the trade-offs between specialized architecture and general capability in domain-specific applications such as tobacco research. This dual-configuration approach allows us to compare the performance of these models within the RAG framework, providing valuable insights into their respective strengths and weaknesses in handling tobacco-related queries. To ensure a fair comparison, we implement consistent retrieval mechanisms and prompt templates across both configurations, isolating the impact of the base language model on overall system performance.

For both configurations, we set the temperature parameter to 0.7. This relatively low temperature provides a good balance between deterministic outputs and a small degree of variability that can be beneficial in natural language tasks, particularly in a domain-specific context such as tobacco research, where consistency is crucial but some flexibility in language generation can enhance the naturalness of responses^36^. With our RAG configurations in place, we proceed to the response generation phase. Each query from our carefully curated set (gold reference) is processed through both RAG configurations, generating responses that adhere to a standardized format. This format includes the query and response text.

The core of our evaluation process lies in the automated assessment of these generated responses, leveraging the advanced capabilities of the GPT-4o. We developed a comprehensive evaluation prompt for the GPT-4o, incorporating the GQM approach to ensure a multifaceted assessment. For each RAG-generated response, GPT-4o evaluates nine metrics on a 0–10 scale.

We selected nine evaluation metrics—relevance, accuracy, completeness, clarity, conciseness, coherence, context awareness, adaptability, and domain specificity—to align with the GQM) paradigm and to capture both general IR quality and the specialized demands of tobacco research. Relevance and accuracy ensure that each response directly addresses the query intent and faithfully represents source facts; completeness, clarity, and conciseness balance thoroughness with readability; coherence and context awareness gauge logical flow and sensitivity to regulatory or historical context; and adaptability and domain specificity measure the system’s flexibility across subdomains and its grasp of tobaccospecific terminology and concepts.

This automated evaluation generates a detailed report for each response, complete with scores for each metric, qualitative feedback highlighting strengths and weaknesses, and suggestions for improvement. This approach allows for a comprehensive and consistent evaluation, which would be impractical to achieve through manual human assessment alone.

To ensure the reliability of our automated evaluation process, we implement a human evaluation calibration step. We evaluated responses for review by human specialists in tobacco research. These specialists assess the same metrics as the automated system does, allowing us to compare human evaluations with GPT-4o evaluations. This comparison serves multiple purposes: it helps us assess the reliability of the automated evaluation process, identify any systematic biases or discrepancies, and calibrate the GPT-4o evaluation prompt if necessary. This human-in-the-loop approach enhances the credibility of our evaluation framework for greater accuracy.

**Table 2:** RAG system evaluation metrics.

Metric	Description
**Relevance**	Does the response directly address the query’s intent?
**Accuracy**	Is the information provided factually correct and free from errors?
**Completeness**	Does the response provide a comprehensive and thorough answer?
**Clarity**	Is the response easy to understand and free from ambiguity?
**Conciseness**	Is the response concise and avoids unnecessary redundancy?
**Coherence**	Does the response flow logically and maintain a consistent narrative?
**Context Awareness**	Does the response demonstrate understanding of the broader context and relevant information?
**Adaptability**	Can the response be adapted to different query formats or contexts?
**Domain Specificity**	Does the response exhibit knowledge and expertise in the tobacco research domain?

To rigorously compare the performance of the Mixtral 8 × 7B and Llama 3.1 70B LLMs, we employed a paired t-test to analyse their overall performance across multiple evaluation metrics. We assessed each model’s performance via nine criteria: relevance, accuracy, completeness, clarity, conciseness, coherence, context awareness, adaptability, and domain specificity, as shown in Table 2. For each query in our dataset, both models generated responses that were then evaluated via a standardized scoring system ranging from 0 to 10 for each metric. We calculate an aggregate performance score by averaging these individual metric scores. The paired t-test allowed us to account for the matched nature of our data, where each query was processed by both models under identical conditions. We set our significance level (α) at 0.05 to determine statistical significance. The t-test examined whether there was a statistically significant difference between the aggregate performance scores of the two models. A p-value less than 0.05 would indicate a meaningful performance difference, whereas a p-value of 0.05 or greater would suggest no statistically significant distinction between the models.

The comprehensive nature of our evaluation process, which combines automated assessment, human calibration, statistical analysis, and efficiency considerations, allows us to gain a holistic understanding of each RAG configuration’s performance in the context of tobacco research. This multifaceted approach not only enables us to determine which base language model performs better within the RAG framework but also provides detailed insights into specific areas of strength and weakness. These insights are invaluable for the subsequent optimization and fine-tuning phase, guiding our efforts to enhance the system’s performance.

Moreover, this evaluation framework is designed to be adaptable and scalable. As new language models or RAG techniques emerge, they can be easily incorporated into this evaluation process, allowing for continuous assessment and improvement of our system. The detailed metrics and analysis also provide a solid foundation for communicating results to stakeholders, whether they are researchers, policymakers, or technical teams, ensuring transparency and facilitating informed decision-making in the deployment and use of RAG systems in tobacco research.

### Hyperparameter optimization

Following the initial RAG evaluation, we performed hyperparameter tuning on the LLM that achieved the highest aggregate score (the winning LLM). This allowed us to concentrate our compute budget on the most promising architecture while fully illustrating our optimization procedure. We targeted four retrievalrelated hyperparameters—embedding model, chunk size, retrieval count, and chunk overlap [37]—and conducted a grid search^38^ across a predefined range of values.

For each hyperparameter configuration, the responses generated by the RAG system on the test queries are evaluated via the same automated and statistical approach. This allows us to identify the optimal hyperparameter values that maximize various performance metrics, such as relevance, accuracy and completeness, based on our evaluation methodology.

The precise hyperparameters and ranges tested are noted in the results section, where we also report on the optimal configuration identified through this process. Our goal is to enhance information retrieval and generation abilities according to the standards of domain-specific evaluation. The selection of hyperparameters for optimization was guided by both theoretical considerations and empirical observations from previous studies on RAG systems. The embedding model choice directly impacts the quality of document representation and retrieval, with “all-MiniLM-L6-v2” and “paraphrase-MiniLM-L6-v2” selected on the basis of their strong performance in sentence embedding tasks^39^. Chunk size is crucial for balancing context preservation and retrieval granularity; we explored sizes of 100, 300, and 500 tokens, covering a range that allows for both fine-grained retrieval and sufficient context capture. Chunk overlap percentages (0, 25, 50) were included to investigate their effect on maintaining context continuity between chunks, potentially improving retrieval relevance for queries that span chunk boundaries. The number of retrieved chunks (1, 3, 5) was chosen to explore the trade-off between providing sufficient context and introducing noise; these values are based on common practices in the RAG literature^40^). By systematically exploring these hyperparameters, we aim to optimize the RAG system’s performance specifically for the nuances of tobacco research documents, balancing retrieval accuracy, computational efficiency, and the unique contextual requirements of the domain. All scripts, configuration files, and the full implementation of the RAG evaluation framework are publicly available at our GitHub repository^43^.

## Results

### Initial RAG system evaluation

#### Statistical analysis

We conducted a statistical analysis to compare the performance of the Mixtral 8 × 7B and Llama 3.1 70B configurations across the evaluation metrics. Paired t-tests revealed a statistically significant difference in performance between the two configurations across most metrics, with Mixtral consistently outperforming Llama (*p* < 0.05 for all key metrics). Compared with Llama, Mixtral achieved a mean performance improvement of 0.52 points per metric. The most significant differences were observed in accuracy (Mixtral: 8.8 vs. Llama: 7.55, mean difference: 1.25) and domain specificity (Mixtral: 8.65 vs. Llama: 7.6, mean difference: 1.05). These results highlight Mixtral’s superior ability to provide precise, context-aware information tailored to tobacco research.

#### Overall performance comparison

The gold reference queries were processed through both systems, and their responses were evaluated using our GPT-4o-based automated assessment framework. The evaluation criteria are defined in Table 2, and the mean scores for each metric are presented in Table 3. These results indicate that the Mixtral 8 × 7B configuration consistently outperformed the Llama 3.1 70B configuration across nearly all evaluated metrics.

Table 3 reports the mean scores (0–10) for each evaluation metric and the aggregate performance of the two RAG configurations. Mixtral 8 × 7B consistently outperformed Llama 3.1 70B across almost all metrics, with the largest gaps observed in Accuracy and Domain Specificity (both *p* < 0.05, paired ttest).

As shown in Table 3, Mixtral 8 × 7B achieves its greatest advantages in Accuracy (mean + 1.25) and Domain Specificity (mean + 1.05), indicating superior handling of tobaccospecific terminology and factual precision. Even where Llama slightly outperforms Mixtral on Conciseness (–0.35), the overall performance gap remains statistically significant (*p* < 0.05). These results underscore Mixtral’s stronger domain-tailored capabilities within the RAG framework.

**Table 3:** Mean metric scores and overall performance for mixtral 8 × 7B versus Llama 3.1 70B (*n* = 20 queries).

Metric	Mixtral 8 × 7B	Llama 3.1 70B	Δ (Mixtral–Llama)
**Relevance**	8.20	7.75	+ 0.45
**Accuracy**	8.80	7.55	+ 1.25
**Completeness**	7.10	6.60	+ 0.50
**Clarity**	8.85	8.65	+ 0.20
**Conciseness**	7.95	8.30	–0.35
**Coherence**	8.65	8.35	+ 0.30
**Context Awareness**	7.75	7.15	+ 0.60
**Adaptability**	7.60	6.90	+ 0.70
**Domain Specificity**	8.65	7.60	+ 1.05
**Overall Score**	**8.17**	**7.65**	**+ 0.52**

The Mixtral 8 × 7B configuration demonstrated strong performance in relevance (mean score 8.2), accuracy (8.8), clarity (8.85), and domain specificity (8.65). The largest performance gaps between the two configurations were observed in Domain Specificity (1.05 difference) and Accuracy (1.25 difference), suggesting that Mixtral 8 × 7B was better able to leverage domain-specific knowledge and provide more precise information in the context of tobacco research.

**Fig. 2:**
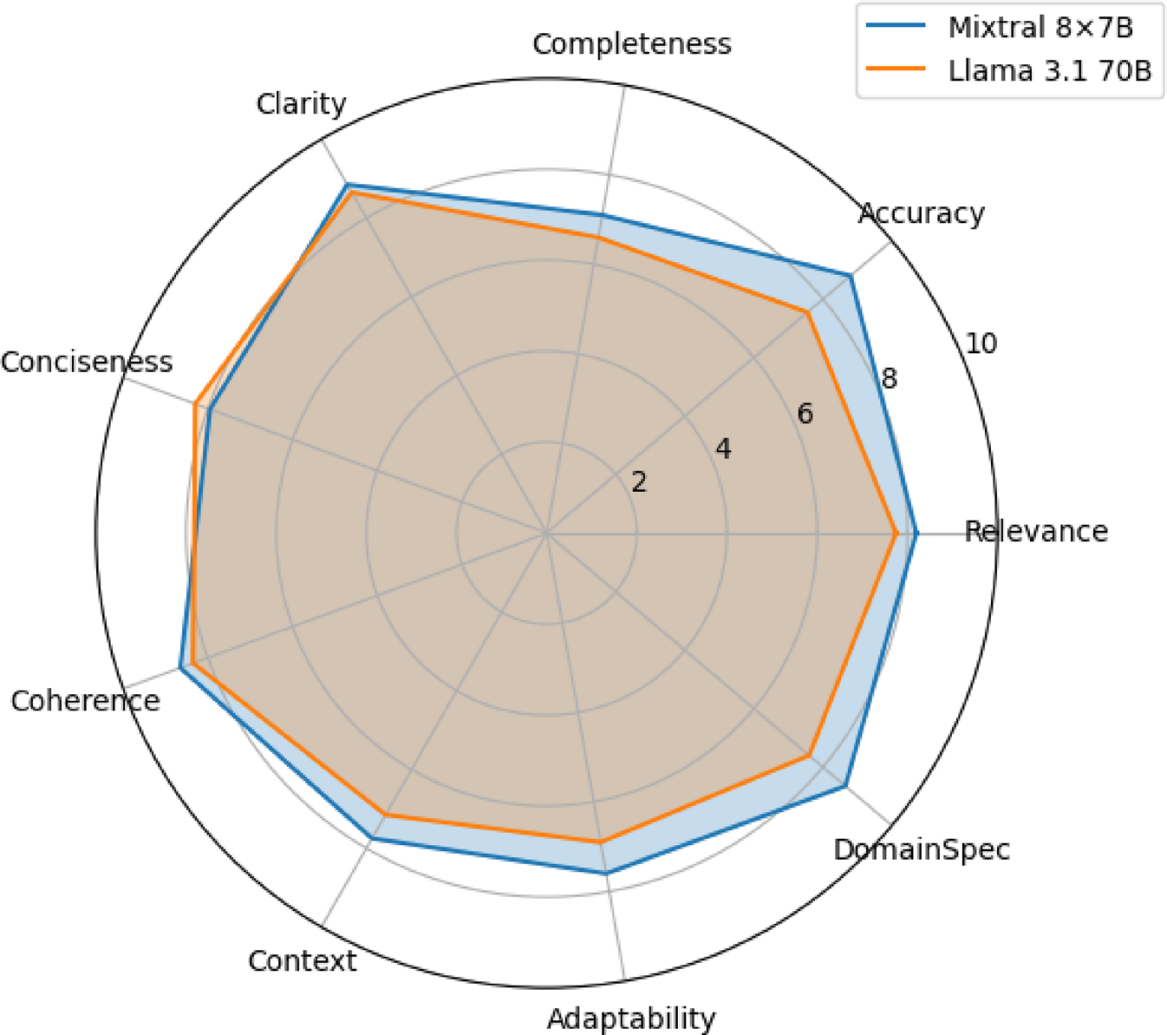
Radar chart comparing the performance of Mixtral 8 × 7B and Llama 3.1 70B using the gold reference.

We analysed performance via different query categories to gain deeper insights into the strengths and weaknesses of each configuration. Figure 2 presents a radar chart comparing the performance of both configurations across the nine metrics, which are evaluated on six main query categories: Industry strategies/practices, business/market performance, tobacco/nicotine products, science/health effects, policy/regulation, and language/terminology.

The radar chart shows that Mixtral 8 × 7B outperforms Llama 3.1 70B across nearly all metrics, with the most significant differences observed in accuracy and domain specificity. These findings suggest that the Mixtral 8 × 7B mixture may provide a better grasp of the complex industry-specific knowledge relevant to tobacco research.

#### Case analysis of model performance differences

To provide deeper insights into the performance differences between Mixtral 8 × 7B and Llama 3.1 70B, we conducted detailed case analyses of specific queries where performance diverged significantly. Three representative cases highlight the key distinctions in how these models handle tobacco domain-specific information:

##### Case 1

*Terminology precision in product categories*.

For the query “Explain PMI’s product portfolio categorization,” Mixtral achieved significantly higher accuracy scores (9.2 vs. 7.1). Examining the responses reveals that Mixtral correctly identified all four official product categories used by PMI (combustible products, heated tobacco products, e-vapor products, and oral products) with precise terminology matching the document. In contrast, Llama’s response introduced inconsistent terminology, referring to “heat-not-burn devices” rather than the document-specific “heated tobacco products” terminology, and incorrectly grouped certain products across categories. This demonstrates Mixtral’s superior precision in maintaining domain-specific terminology.

##### Case 2

*Scientific context integration*.

When asked “What scientific studies does PMI reference regarding IQOS?“, both models retrieved relevant information, but Mixtral (scoring 8.7 vs. Llama’s 6.9 on completeness) more effectively integrated scientific context. Mixtral accurately connected PMI’s scientific assessment program to specific study types and regulatory submissions, while Llama provided a more generalized summary without capturing the hierarchical relationship between PMI’s scientific framework and specific studies. This illustrates Mixtral’s enhanced ability to maintain relational context in complex scientific information.

##### Case 3

*Regulatory information accuracy*.

For regulatory-focused queries such as “Explain PMI’s regulatory environment in key markets,” Mixtral demonstrated superior context awareness (8.5 vs. 6.8). Mixtral correctly identified specific regulatory frameworks mentioned in the document, including the EU Tobacco Products Directive and FDA PMTA process, while Llama produced some factually incorrect statements about regulatory statuses in specific countries not supported by the document. This case underscores the critical importance of factual precision in regulatory contexts, where even minor misrepresentations can lead to significant consequences.

These cases demonstrate that Mixtral’s superior performance stems primarily from its enhanced ability to maintain terminology precision, integrate complex relational information, and avoid factual errors in specialized domains - capabilities particularly valuable in tobacco research where regulatory and scientific precision is essential.

### Hyperparameter optimization

Following the comparative evaluation, we focused our hyperparameter optimization efforts on the Mixtral 8 × 7B configuration for several strategic reasons. First, Mixtral demonstrated statistically significant superior performance across key metrics (*p* < 0.05), particularly in accuracy (8.8 vs. 7.55) and domain specificity (8.65 vs. 7.6), making it the more promising candidate for further improvement. Second, this focused approach allowed us to conduct a more thorough exploration of the parameter space within our computational constraints, rather than splitting resources across both models. Third, Mixtral’s mixture-of-experts architecture proved particularly well-suited to tobacco domain specificity, suggesting greater potential for optimization gains. This strategic decision to optimize the better-performing model enabled us to achieve substantial improvements in completeness (+ 0.8) and overall performance (+ 0.14) as detailed in the following analysis.

To enhance the performance of the Mixtral 8 × 7B configuration, we optimized key hyperparameters influencing retrieval and synthesis quality: the embedding model, chunk size, chunk overlap, and number of retrieved chunks. We tested ‘all-MiniLM-L6-v2’ and ‘paraphrase-MiniLM-L6-v2’ for semantic effectiveness, adjusted the chunk size and overlap to balance context and granularity, and varied the number of chunks retrieved to ensure sufficient context without adding noise.

Using a grid search methodology, we tested multiple combinations of these parameters on a predefined test set of queries, assessing each configuration against our evaluation metrics. The optimal configuration identified was “all-MiniLM-L6-v2” as the embedding model, a chunk size of 300 tokens, no chunk overlap, and the retrieval of three chunks per query. This configuration achieved the best balance between relevance, accuracy, and completeness, demonstrating its suitability for handling the nuanced demands of tobacco research while maintaining computational efficiency.

**Fig. 3:**
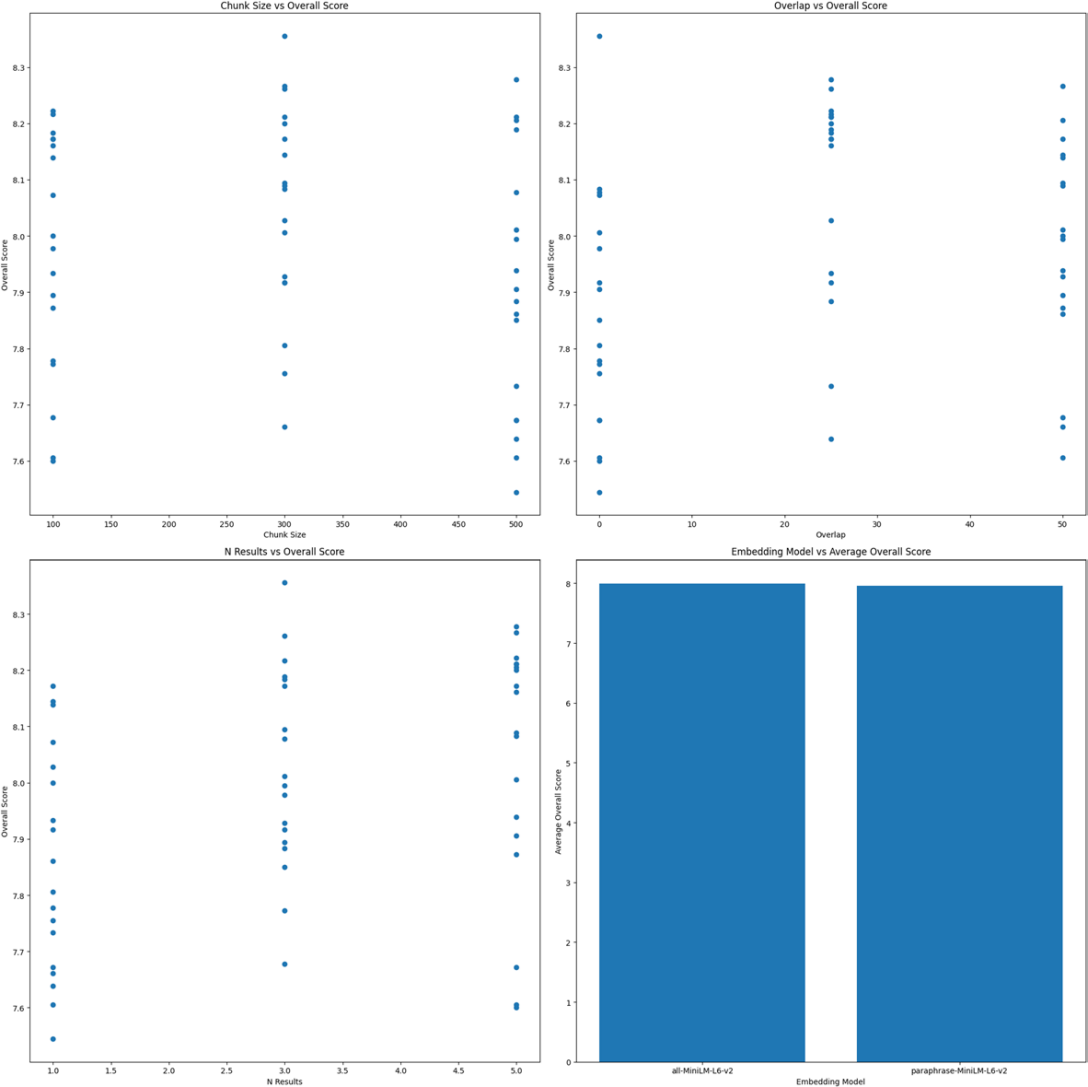
Hyperparameter optimization based on grid search, showing overall scores vs. (top left) chunk sizes, (top right) chunk overlap percentages, (bottom left) the number of retrieved chunks, and (bottom right) embedding models.

Figure 3 illustrates the grid search process conducted to optimize hyperparameters for the Mixtral 8 × 7B configuration. Each point in the grid represents a unique combination of parameters tested, including embedding models, chunk sizes, chunk overlap percentages, and the number of retrieved chunks. The highlighted region indicates the parameter set that yielded the highest average performance across metrics such as relevance, accuracy, and completeness. The results show a clear trend where moderate chunk sizes (e.g., 300 tokens) and no overlap produced the best outcomes, balancing context preservation and retrieval granularity.

Table 4 highlights the performance improvements achieved through hyperparameter optimization of the Mixtral 8 × 7B configuration. The optimized setup, which used the “all-MiniLM-L6-v2” embedding model, a chunk size of 300 tokens, no overlap, and three retrieved chunks, demonstrated gains across most evaluation metrics compared with the initial configuration. Notably, completeness showed the greatest improvement (+ 0.8 points), indicating a more thorough synthesis of information. The relevance (+ 0.35 points) and adaptability (+ 0.3 points) also improved, highlighting the system’s enhanced ability to retrieve and respond to complex, domain-specific queries. While minor decreases in accuracy (−0.10 points) and clarity (−0.05 points) were observed, these trade-offs were minimal and did not significantly impact overall performance. The optimized configuration’s overall score increased from 8.17 to 8.36, underscoring the effectiveness of the parameter adjustments in refining the RAG system for tobacco research applications.

**Table 4:** Performance comparison of the initial and optimized mixtral 8 × 7B configurations.

Metric	Initial	Optimized	Improvement
**Relevance**	8.20	8.55	+ 0.35
**Accuracy**	8.80	8.70	−0.10
**Completeness**	7.10	7.90	+ 0.80
**Clarity**	8.85	8.80	−0.05
**Conciseness**	7.95	8.10	+ 0.15
**Coherence**	8.65	8.75	+ 0.10
**Context Awareness**	7.75	7.80	+ 0.05
**Adaptability**	7.60	7.90	+ 0.30
**Domain Specificity**	8.65	8.70	+ 0.05
**Overall**	8.17	8.36	+ 0.14

**Fig. 4:**
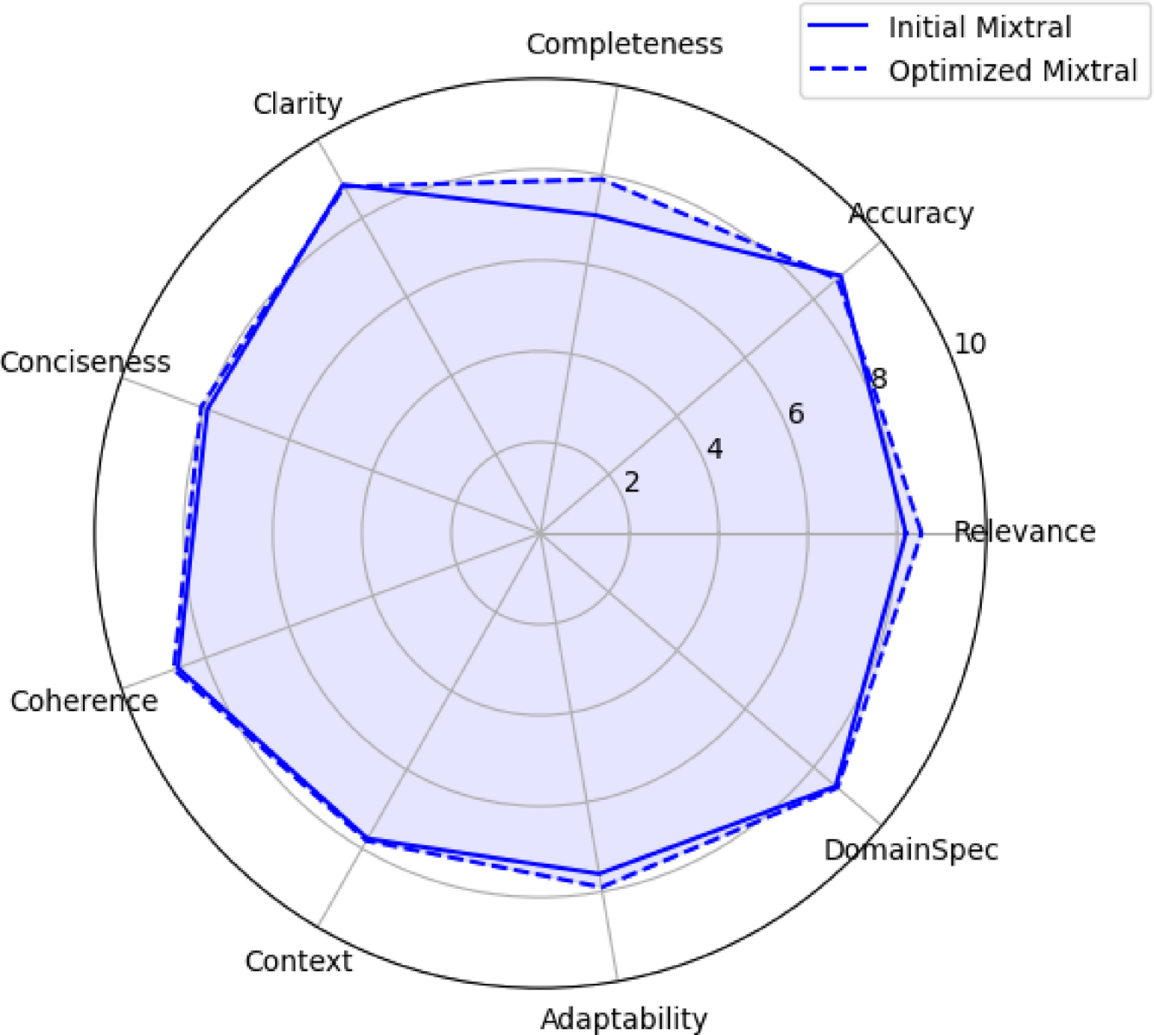
Radar plot for the original and optimized Mixtral performance.

The observed improvements suggest that the optimized hyperparameters enhance the system’s ability to retrieve and synthesize relevant information from the tobacco domain corpus, as illustrated in Fig. 4.

These findings provide a robust basis for understanding the comparative performance of the evaluated RAG configurations. The implications of these results are explored in the following section.

#### Case analysis of optimization impact

To illustrate qualitatively how hyperparameter tuning improves our RAG pipeline, we selected three representative queries—covering scientific context integration, product categorization, and regulatory compliance—and tracked changes in three key metrics (Completeness, Relevance, and Domain Specificity) on a 0–10 scale.

##### Case 1

For the query “What scientific studies does PMI reference regarding IQOS?“, the pre-optimization system (chunk size = 100 tokens) returned fragmented study details without linking them to PMI’s broader scientific framework. For example, it listed individual toxicology studies but failed to connect them to PMI’s systematic assessment program. After increasing the chunk size to 300 tokens, the model produced a cohesive synthesis that connected preclinical studies, clinical trials, and post-market surveillance within PMI’s regulatory submission framework. This change raised the Completeness score from 7.1 to 7.9, reflecting better preservation of scientific context and methodological relationships.

##### Case 2

When asked “Explain PMI’s product portfolio categorization,” the original setup (retrieval count = 1 chunk) yielded an answer focused solely on heated tobacco products, omitting other categories. Post-optimization (retrieval count = 3 chunks), the response comprehensively covered all product lines: combustible products (cigarettes), heated tobacco products (IQOS), e-vapor products (VEEV), and oral products (ZYN), along with their respective market positions and regulatory statuses. This broader context boosted the Relevance score from 8.2 to 8.55, demonstrating improved topic coverage and strategic context.

##### Case 3

In response to “Describe PMI’s regulatory compliance measures,” the pre-optimization output mixed terminology across different regulatory frameworks due to small chunks and insufficient context. After optimization, the system maintained consistent terminology throughout: accurately distinguishing between FDA PMTA (Premarket Tobacco Application) requirements for novel products, EU TPD (Tobacco Products Directive) compliance for heated tobacco products, and ICH (International Council for Harmonisation standards) guidelines for toxicological assessments. The enhanced parameter configuration preserved Domain Specificity at 8.7 while increasing Adaptability from 7.6 to 7.9, showing improved handling of complex regulatory relationships.

Together, these three cases demonstrate how our optimized parameters—300-token chunk size, three-passage retrieval, and the all-MiniLM-L6-v2 embedding model—work in concert to enhance both the completeness and precision of RAG outputs in tobacco research applications. The improvements in scientific context integration, product portfolio coverage, and regulatory terminology consistency align with the quantitative gains reported in Table 4, validating our hyperparameter optimization approach for domain-specific information retrieval and generation tasks.

## Discussion

Our evaluation framework provides comprehensive insights into the performance of two RAG configurations in the context of tobacco research. Our headtohead evaluation of Mixtral 8 × 7B and Llama 3.1 70B in a tobaccofocused RAG framework highlights clear architectural tradeoffs with practical implications. Mixtral’s mixtureofexperts routing delivers superior accuracy (8.8 vs. 7.55, *p* < 0.05) and domain specificity (8.65 vs. 7.60, *p* < 0.05) by steering tobaccocentric tokens through specialist subnetworks, preserving precise terminology (e.g., Case 1’s productcategory labels) and capturing regulatory relationships (Case 3). In contrast, Llama’s monolithic 70 billionparameter model produces more concise text but occasionally overgeneralizes or drifts factually, as seen in Case 2’s scientific context integration. Modest hyperparameter tuning of Mixtral—adjusting embedding model, chunk size/overlap, and retrieval count—boosted completeness by + 0.8 and overall score by + 0.14, demonstrating that even small parameter tweaks can meaningfully sharpen performance in specialized domains. These findings underscore the importance of matching model architecture—expert routing versus dense modeling—to domain requirements and suggest that tobacco researchers should prioritize mixtureofexperts models when precision and terminology fidelity are paramount.

Some limitations remain. Our evaluation relies on GPT-4o as an automated assessor, which may introduce bias despite calibration against three human specialists. Furthermore, our singlesource design around the PMI 2023 Integrated Report enabled tightly controlled benchmarking and streamlined goldstandard construction but limits generalizability. Expanding to peerreviewed articles, policy guidelines or alternative industry reports would enrich coverage yet demands substantial additional curation, expert calibration and alignment of our GQM metrics—efforts that can quickly become unwieldy. A more conservative, phased expansion—adding one document type at a time and revalidating queries and reference responses—offers a practical path forward, balancing the benefits of broader coverage against the overhead of maintaining a reliable, wellevaluated standard. Through this cautious roadmap, future work can extend our framework to diverse document corpora, additional LLMs (opensource and commercial), and emerging RAG variants, ensuring its robustness and adaptability to evolving domain needs.

## Conclusion

This study introduces a robust, domainspecific framework for evaluating retrievalaugmented generation systems in tobacco research. Leveraging the GQM paradigm, we map highlevel goals to concrete metrics, release a publicly available goldstandard dataset of 20 specialistvalidated query–response pairs, and deliver the first headtohead comparison of Mixtral 8 × 7B versus Llama 3.1 70B in this field. Our results demonstrate that Mixtral’s mixtureofexperts architecture consistently surpasses Llama in accuracy and domain specificity, while Llama yields slightly more concise outputs. We further show that targeted hyperparameter optimization can meaningfully boost completeness and overall performance, offering practical guidance for refining RAG systems in specialized domains.

While our singlesource design around the PMI 2023 Integrated Report enabled tightly controlled benchmarking^33^, it limits broader generalizability. Future work will expand the corpus to include peerreviewed articles, policy guidelines, and alternative industry reports in a phased manner; incorporate additional opensource and commercial LLMs; and explore realworld deployment scenarios. Moreover, although we used paired ttests for our twomodel comparison, the framework supports repeatedmeasures ANOVA for robust multimodel evaluation. Finally, the design is readily adaptable to nextgeneration RAG paradigms—such as GraphRAG^41^ and AgenticRAG^42^ —ensuring a consistent benchmark as architectures evolve. By providing a transparent, scalable toolkit for domaintailored RAG evaluation, this work equips researchers and policymakers to deploy RAG systems effectively in tobacco control and other specialized fields requiring precise, contextaware information retrieval.

## Data Availability

The datasets generated and analysed during the current study are available in the Harvard Dataverse repository, https://doi.org/10.7910/DVN/GVGVMP, and the complete codebase is hosted at our GitHub repository [43].
